# What Is the Quality of Life in Patients Treated with Levothyroxine for Hypothyroidism and How Are We Measuring It? A Critical, Narrative Review

**DOI:** 10.3390/jcm10071386

**Published:** 2021-03-30

**Authors:** Françoise Borson-Chazot, Jean-Louis Terra, Bernard Goichot, Philippe Caron

**Affiliations:** 1 Federation d’Endocrinologie, Hôpital Louis Pradel, Hospices Civils de Lyon, 69677 Bron, France; 2 Research on Healthcare Performance (RESHAPE), INSERM U1290, Université Claude Bernard Lyon 1, Domaine Rockefeller, 69003 Lyon, France; 3 Association pour la Promotion des Techniques Nucléaires en Médecine et en Biologie, 69003 Lyon, France; 4 EA 4129 Parcours Santé Systémique P2S Université Claude Bernard Lyon 1, 69100 Villeurbanne, France; jl_terra@hotmail.com; 5 Service de Médecine Interne, Endocrinologie et Nutrition, Hôpital de Hautepierre, Hôpitaux Universitaires de Strasbourg, 67200 Strasbourg, France; bernard.goichot@chru-strasbourg.fr; 6 Service d’Endocrinologie, Maladies Métaboliques et Nutrition, Pôle Cardio-Vasculaire et Métabolique, Hôpital Larrey, CHU de Toulouse, 31059 Toulouse, France; caron.p@chu-toulouse.fr

**Keywords:** levothyroxine, LT4, autoimmune thyroiditis, quality of life, combination therapy, LT3

## Abstract

Thyroid hormone replacement therapy (THRT, generally using oral levothyroxine (LT4)) is a safe, effective means of treating hypothyroidism. However, a proportion of LT4-treated patients with biochemically normal thyroid function tests complain of persistent symptoms that impact their health-related quality of life (QoL). The objectives of this critical, narrative review of the literature were to identify studies of QoL in LT4-treated patients with hypothyroidism, examine the instruments used to measure QoL, determine whether normal QoL is restored by THRT, and identify factors associated with QoL. The PubMed database was searched from 1 January 2000 to 31 December 2020. A total of 809 publications were screened, 129 full-text articles were retrieved, and 58 were analyzed. The studies of overt hypothyroidism evidenced an improvement in psychological and emotional well-being after three to six months of THRT with LT4, although contrasting results were found for patients with subclinical hypothyroidism. Combination treatment with LT4 and liothyronine was not generally associated with better QoL. In hypothyroidism, QoL appears to be influenced by a number of physiological, behavioral, cognitive and/or lifestyle factors that are not strictly related to thyroid hormone levels.

## 1. Introduction

Hypothyroidism (mainly caused by autoimmune thyroiditis, surgery for cancer or benign thyroid disease, or radioiodine therapy) impacts many physiological functions [1]. Although the definition and prevalence of hypothyroidism depend on biochemical reference ranges measured in the general population, it has been estimated that ~4% of adults in Western countries suffer from overt hypothyroidism [2,3]. The prevalence rises to ~15% when subclinical hypothyroidism (SCH, defined as a serum thyroid-stimulating hormone (TSH) level above the upper limit of normal (typically >4.0 mU/L) and a normal serum free thyroxine (FT4) level) is included [4,5,6].

Thyroid hormone replacement therapy (THRT, generally based on oral levothyroxine (LT4)) is a safe, effective means of treating hypothyroidism [7,8,9,10,11]. The clinical objective is to relieve symptoms and then normalize thyroid hormone levels. TSH is considered to be the most sensitive and specific marker of thyroid status [12]. Most LT4-treated patients experience fewer symptoms of hypothyroidism at a serum TSH level of between 0.4 and 4.0 mU/L. Nevertheless, a small proportion of LT4-treated patients (around 5 to 10%, depending on the study) complain of persistent symptoms of hypothyroidism, mood disturbances, and poor health-related quality of life (QoL) even when thyroid function is biochemically normal [13,14,15].

Well-being and QoL do not simply equate to adequate material living standards or the absence of disease. QoL has been defined as how an individual’s well-being is affected over time by a disease, disability, or disorder [13,14,15] but can also be viewed as the difference between an individual’s hopes and his/her actual status [16]. Hence, QoL is a subjective variable that covers affective, cognitive, and behavioral domains [17,18]. This subjectiveness means that inter-individual and inter-group comparisons of QoL (e.g., between actively treated and placebo-treated patients or with general population samples) are problematic [19]. Researchers have sought to codify QoL with psychometrically valid generic or disease-specific instruments. The fact that a large number of QoL instruments have been developed reflects the complexity of this variable and the differences in its definition.

The objectives of this critical, narrative review of the literature were to (i) identify studies of QoL in LT4-treated adults and children with hypothyroidism (including SCH), (ii) examine the instruments used to measure QoL in the identified studies, (iii) determine whether normal QoL is restored by THRT, and (iv) identify physiological, genetic, demographic and behavioral factors associated with QoL in patients with hypothyroidism. To this end, we searched the literature for quantitative reports on QoL in patients undergoing THRT with LT4 alone or in combination with other thyroid hormones. We focused on the results of randomized clinical trials (RCTs) but did not exclude nonrandomized studies.

## 2. Summary of the Literature Search

The PubMed database was searched from 1 January 2000 to 31 December 2020, using combinations of the following terms (in English only): hypothyroid*, “quality of life”, “QoL”, “L-T4”, “LT4”, “LT3”, “T3”, liothyron*, levothyrox*, “thyroid hormone deficiency”, “thyroid hormone replacement”, “THRT”, “ThyDQoL”, “ThyPRO”, “Short-Form 36”, and “SF-36”. The PubMed search query was ((“2000/01/01”[Date-Publication]: “3000”[Date-Publication])) AND ((LT4 OR L-T4 OR LT-3 OR LT3 OR T3 OR “thyroid hormone replacement” OR “THRT” OR levothyrox* OR liothyron* OR “thyroid hormone deficiency” OR “hypothyroid*”) AND (“quality of life” OR “wellbeing” OR “well-being” OR “satisfaction” OR “mood” OR “SF-36” OR QoL OR “short-form 36” OR “SF36” OR “SF36” OR ThyPRO)).

All authors discussed and decided on the study inclusion and exclusion criteria. Only publications that reported numerical QoL data on patients receiving THRT for benign overt hypothyroidism or SCH were included. We excluded studies of patients with differentiated thyroid carcinoma (DTC) because their baseline status and TSH targets differ from those in benign hypothyroidism [20] and because a diagnosis of cancer may be associated with poor QoL for psychological reasons [21,22]. Lastly, we excluded case reports, reviews, and editorials. All abstracts were screened for relevance by the lead author (FBC). If an abstract was found to be relevant, the full-text article was retrieved and reviewed. The full-text articles for review were divided between all the authors, who extracted the data. The lead author checked the extracted data. Any disagreements were resolved by consensus.

**Figure 1:**
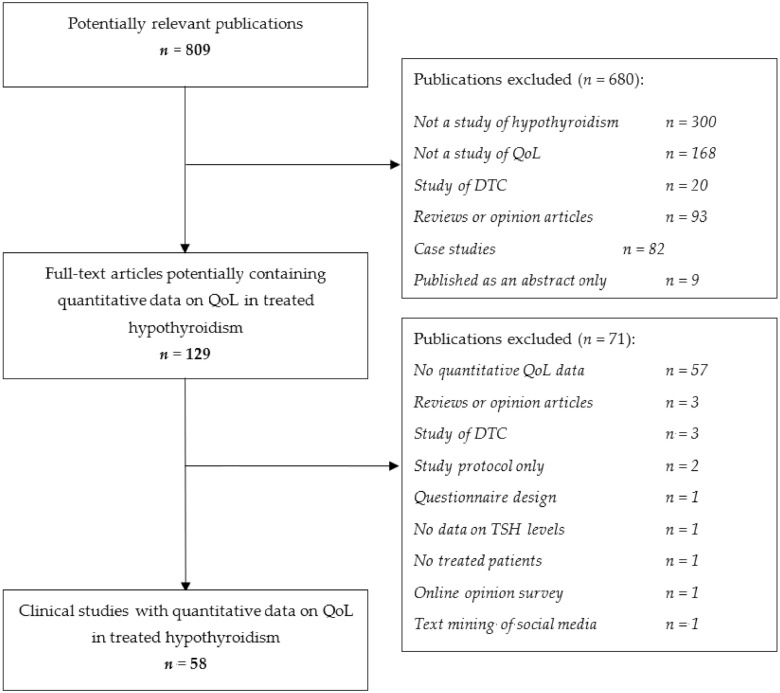
Study selection flow chart. The PubMed database was searched from 1 January 2000 through 31 December 2020. QoL: quality of life; TSH: thyroid-stimulating hormone.

The PubMed search initially identified a total of 809 potentially relevant publications (Figure 1).

The main reasons for excluding publications were (i) the absence of original, quantitative data on QoL, and (ii) the absence of data on patients with hypothyroidism. A total of 129 full-text articles were retrieved; 58 of these met the inclusion criteria and were analyzed. It should be noted that we did not seek to determine independently whether the QoL reported for a given study group was truly “good” or “normal”; we relied on the study investigators’ statements and the results of their statistical analyses.

## 3. Quality of Life in Patients Treated for Hypothyroidism

### 3.1. How Has QoL Been Measured in Patients on THRT for Hypothyroidism?

When considering the selected studies, we noted the use of 12 generic QoL questionnaires and five disease-specific QoL questionnaires. The questionnaires’ characteristics are summarized in Table 1.

The most frequently applied generic instrument was the well-known Short Form (36) Health Survey (SF-36) [31]. The five thyroid-specific instruments were the Underactive Thyroid-Dependent Quality of Life Questionnaire (ThyDQoL) [23], the Thyroid-Specific Patient-Reported Outcome Measure (ThyPRO) [24], the Hypothyroid-Specific Health-Related Quality of Life Questionnaire (HRQL) [25], and the Underactive Thyroid Treatment Satisfaction Questionnaire (ThyTSQ) [26] and a modified version of the Chronic Thyroid Questionnaire [28]. Although the ThyTSQ probes treatment satisfaction, the investigators of a recent study referred to it as a “dedicated QoL questionnaire”; hence, we included the ThyTSQ in our list of disease-specific instruments [44]. A systematic review has recommended the ThyPRO for administration in benign thyroid diseases [45]. It should be noted that QoL questionnaires often comprise “physical” items (which may overlap with symptoms, for example) and “mental” items (which may overlap with mood, for example). For example, the SF-36 features a physical composite score (PCS) and a mental composite score (MCS) calculated from the individual section scores, using a proprietary algorithm [31]. These considerations illustrate the complexity of measuring (and distinguishing between) specific symptoms, nonspecific symptoms, QoL, treatment satisfaction, and patient preference.

Fifty of the 58 reviewed publications featured a single QoL instrument, and eight featured two instruments (Table 2 and Table 3).

Lastly, it should be borne in mind that QoL was rarely the primary study outcome, and so many of the interventional trials were not powered to reveal a putative effect on this variable.

### 3.2. In Patients with Overt Hypothyroidism, Does LT4 Treatment Result in Normal QoL?

#### 3.2.1. Randomized Studies

We found very few RCTs of patients treated with LT4 soon after being diagnosed with overt hypothyroidism (Table 2). This is not surprising because newly diagnosed patients require effective treatment and thus (for ethical reasons) cannot be put on a placebo. However, Samuels et al. studied a high-dose LT4 group vs. a standard-dose (control) group in a double-blind cross-over RCT [55]. The intergroup differences in several SF-36 domains were inconsistent; at the end of the study, the mean ± SEM score in the high-dose group was significantly higher for the PCS (*p* = 0.01) but not for the MCS (*p* = 0.15). The changes in the MCS (but not in the PCS) were associated with changes in FT3 and FT4 levels. In a double-blind crossover trial, Bolk et al. (2010) randomized adult patients to morning or evening LT4 administration for 3 months but did not compare the QoL scores with controls or with normative data. QoL improved significantly over time but there were no significant intergroup differences in the SF-36 domain scores [58].

#### 3.2.2. Nonrandomized Studies

The great majority of studies of patients newly treated with LT4 following a diagnosis of overt hypothyroidism had observational designs (Table 3). Two studies found that QoL was normal (i.e., not impaired, relative to general population samples). In a study of German children and adolescents with various thyroid diseases, Hirtz et al. observed a normal KINDL-R score in the subsamples of participants with overt hypothyroidism or SCH [91], and Quinque et al. (2013) did not find a difference in the ThyDQoL score when comparing treated patients with autoimmune thyroiditis and a group of healthy controls [69]. Other nonrandomized studies found small but statistically significant impairments in QoL (using SF-36 and/or the ThyPRO) among LT4-treated patients with overt hypothyroidism, relative to general population samples [68,92]. Lastly, three studies reported significantly worse QoL in LT4-treated patients with overt hypothyroidism, relative to control patients with other diseases and to general population samples ([66,73,86].

#### 3.2.3. Summary

Overall, the literature results suggest that QoL is markedly improved-but not always normalized-by several months of LT4 treatment in newly diagnosed patients with overt hypothyroidism. The sometimes conflicting or inconsistent findings might be due to major interstudy differences in designs, patient populations, and control populations. The absence (for ethical reasons) of control (i.e., non-treated) groups of patients newly diagnosed with overt hypothyroidism means that the full effect of LT4 on QoL might be underestimated.

### 3.3. Is LT4 + LT3 Combination Treatment Associated with Normal QoL in An Indication of Overt Hypothyroidism?

#### 3.3.1. Randomized Studies

Some LT4-treated patients have relatively low serum T3 levels, i.e., an abnormally high, nonphysiological T4/T3 ratio that might be related to abnormally low T3 secretion by the thyroid or low peripheral T3 production (due to decreased deiodinase activity in depression or in metabolic diseases) [94,95,96,97]. From the early 2000s onwards, it has often been suggested that patients who experience persistent symptoms of hypothyroidism and/or poor QoL during treatment with LT4 alone may benefit from LT4 + LT3 combination treatment. However, almost 20 years later, the merits and limitations of this strategy are still subject to debate [97,98,99].

In most of the double-blind randomized studies, LT4 + LT3 combination therapy was not associated with better QoL (vs. LT4 alone) (Table 2). This was true in Clyde et al.’s (2003) double-blind RCT (using a custom hypothyroid-specific health-related quality of life questionnaire) [25], Sawka et al.’s (2003) double-blind RCT (using the 10-domain Medical Outcomes Study (MOS) [47]), Walsh et al.’s (2003) double-blind, crossover RCT (using the SF-36) [48], Escobar-Morreale et al.’s (2005) double-blind crossover RCT [50], Appelhof et al.’s (2005) study of LT4, LT4:LT3 in a 10:1 ratio, and LT4:LT3 in a 5:1 ratio (using the RAND-36) [51], and Kaminski et al.’s (2016) double-blind crossover RCT using a health-related QoL questionnaire based on the Chronic Thyroid Questionnaire [27]. Similarly, Hoang et al.’s (2013) double-blind, crossover RCT (using the 12-item General Health Questionnaire (GHQ-12)) did not find an advantage for dried thyroid extract (which contains variable, non-standardized amounts of T3) over LT4 [60]. Nygaard et al.’s (2009) double-blind crossover RCT reported significant intergroup differences for the SF-36 General Health and Vitality domains but not for the Social functioning and Mental Health domains [56].

#### 3.3.2. Nonrandomized Studies

A few nonrandomized studies found that some QoL benefits were associated with LT4 + LT3 combination therapy (Table 3). In an open-label longitudinal cohort study of a switch to LT4 + LT3, Michaelsson et al. (2018) reported that the ThyPRO-39 score fell (indicating a reduction in symptoms of hypothyroidism and better QoL) from 54 [34,35,36,37,38,39,40,41,42,43,44,45,46,47,48,49,50,51,52,53,54,55,56,57,58,59,60,61,62,63,64,65,66,67,68,69,70,71,72,73,74] at baseline to 15 [11,12,13,14,15,16,17,18,19,20,21,22,23,24,25,26,27,28] after 3 months (*p* < 0.0001) and then to 20 [14,15,16,17,18,19,20,21,22,23,24,25,26] after 12 months [82]. The baseline ThyPRO-39 composite score was correlated with the baseline free T3 (FT3) level (*p* = 0.02). In fact, this finding argued against the hypothesis whereby high FT3 levels are essential for good QoL [82]. Tariq et al. (2018) found an advantage in the MOS SF-20 score in a retrospective study of a switch from LT4 monotherapy to LT4 + LT3 combination therapy or dried thyroid extract [83]. Lastly, in an open-label, single-arm study, Jonklaas and Burman (2016) documented a significant rise in the SF-36 PCS but not in the MCS after 4 and 5 weeks of treatment with a daily 30–45 µg dose of LT3 [76]. The investigators hypothesized that dissatisfaction with a hormone therapy regimen might be related to fluctuations in peak and trough serum T3 and FT3 levels.

#### 3.3.3. Summary

Most studies of combination therapy did not evidence a difference in QoL for LT4 + LT3 combination therapy, relative to LT4 alone [25,47,48,50,51,60]. When benefits were found, they concerned some but not all QoL domains [56,82,83]. The majority subjective patient preference for LT4 + LT3 combination therapy (typically expressed by ~50–70% of the treated patients [50,51,56]) cannot be explained by the QoL data per se. The basis for this patient preference has yet to be determined. As discussed below, we hypothesize that today’s QoL instruments do not necessarily capture subtle changes (e.g., in mood) related to patient preference and wellbeing. Lastly, the presence of an additional placebo effect (perhaps due to the novelty of taking a combination treatment after months or years of taking LT4 alone) cannot be ruled out.

### 3.4. Is THRT Associated with Normal QoL in an Indication of SCH?

#### 3.4.1. Randomized Studies

In contrast to treated overt hypothyroidism, the relationship between LT4 treatment and QoL in SCH has been frequently evaluated in double-blind, placebo-controlled RCTs (Table 2). In a study of LT4 (a fixed 100 µg/day) vs. placebo, Razvi et al. (2007) studied 100 previously untreated patients [median (range) TSH level at baseline: 5.3 mU/L (3.7–15.8)] [53]. The mean ± SD ThyDQoL scores in the LT4 and placebo phases did not differ significantly. In a double-blind study performed in Brazil, Reuters et al. (2012) assigned treatment-naïve patients with SCH to either LT4 (*n* = 35) or placebo (*n* = 36) for 6 months [59]. In the LT4 group, the Role-emotional and Pain SF-36 scores improved markedly over time and differed significantly from placebo. In the large, double-blind, randomized Thyroid Hormone Replacement for Subclinical Hypothyroidism (TRUST) study of community-dwelling patients aged ≥ 65 with SCH, an association between treatment and improved thyroid-specific QoL (as measured with the ThyPRO) or general QoL (as measured with the EuroQol five-dimensional questionnaire (EQ-5D)) was not observed [65,100]. Mooijaart et al. (2019) studied pooled data from patients aged 80 and over with SCH in the Institute for Evidence-Based Medicine in Old Age (IEMO) and TRUST RCTs [88]. The few small changes in the mean ± SD ThyPRO subscores were not statistically significant. Lastly, Pollock et al. performed a double-blind, placebo-controlled RCT of LT4-treated patients with symptoms of hypothyroidism but who were biochemically euthyroid; there were no significant intergroup differences in any of the QoL (SF-36) scores [46].

#### 3.4.2. Nonrandomized Studies

The results of nonrandomized studies [either cross-sectional or longitudinal (Table 3)] were disparate: some studies found no significant differences in QoL between treated patients and healthy controls [69,101], others observed better scores in some but not all QoL domains in treated patients [13,68,84,89], and yet others reported the presence or persistence of significantly worse QoL in treated patients [23,58,66,73,86].

#### 3.4.3. Summary

With a few exceptions, studies of patients with SCH indicate that LT4 treatment does not markedly improve QoL and that the latter is not correlated with thyroid hormone concentrations. However, age might be a factor. As mentioned below, older age is [inconsistently] associated with poor QoL. It is possible that in older adults with comorbidities, today’s QoL instruments are not sensitive enough to measure subtle effects of changes in LT4. Studies of QoL in younger adults with SCH are warranted.

### 3.5. Which Factors Might Influence or Determine QoL in Patients on THRT with Hypothyroidism?

#### 3.5.1. TSH Levels

Several groups have looked at whether steady-state or target TSH levels/LT4 dose levels in treated patients are correlated with QoL [102]. In an observational study and an RCT, Samuels et al. found that high-normal (>2.5 mU/L) vs. low-normal (≤2.5 mU/L) TSH levels [75] or low normal, high-normal, and mildly elevated target TSH levels [81] did not significantly influence the SF-36 MCS and PCS scores and did not differ significantly, apart from a higher Physical Functioning score in the high-normal TSH group. Dos Santos Vigario et al.’s cross-sectional study (2013) of 2057 LT4-treated patients with primary hypothyroidism [70] concluded that only “undertreatment” (TSH > 4.0 mU/L) was associated with poor SF-36 scores. A similar lack of influence of the target TSH level was obtained in Roos et al.’s (2005) double-blind RCT [49] and in Walsh et al.’s crossover RCT (69). In contrast, Moron-Diaz et al. found that even within the TSH reference range, lower TSH levels were associated with better QoL with adequately treated primary hypothyroidism [93]. Similarly, Mithal et al. (2014) observed that the mean SF-36 Emotional Health and Physical Health scores were worse (*p* = 0.0278 and 0.0763, respectively) among “undertreated” patients (TSH > 4 mU/L) and “overtreated” patients (TSH < 0.40 mU/L) patients when compared with patients with a normal TSH level [71]. In view of these studies’ sometimes conflicting conclusions, the exact nature of the relationship between QoL and steady-state or target TSH levels is still unclear and requires further investigation.

#### 3.5.2. Genetic Factors (Transport Proteins and Metabolic Enzymes)

In view of the possible importance of differences or fluctuations in plasma and/or tissue FT3 levels on symptoms and QoL, a few research groups have genotyped patients for the genes coding for deiodinases or for proteins that transport thyroid hormones in vivo. Van der Deure et al. (2008) screened patients for polymorphisms in the OATP1C1 gene coding for the brain-specific thyroid hormone transporter called organic anion transporting polypeptide 1C1 [103]; there were a few significant differences in the RAND-36 score (e.g., for the rs10770704 variant), with the greatest difference seen for homozygotes. In LT4-treated individuals from the LifeLines cohort study, Wouters et al. (2017) did not find an association between poor QoL (RAND-36) and the rs225014 Thr92Ala genotype of the DIO2 gene coding for deiodinase (DIO) 2 [79]. After adjustment for age and sex, Young Cho (2017) found significantly lower median SF-36 PCS scores in hypothyroid patients who were homozygous for two DIO1 variants (rs11206244 and rs2294512) [78]. By scoring the GHQ-12 in LT4-treated patients, Panicker et al. found that the CC genotype of the rs225014 polymorphism in the DIO2 gene was associated with impaired psychological well-being and a better response to combination therapy [57].

#### 3.5.3. Other Factors

Symptoms of hypothyroidism influence QoL, as shown by Tan et al.’s (2019) open-label, cross-sectional survey. 229 LT4-treated patients were divided into groups with below-median (<0.78) or above-median EQ-5D questionnaire scores [90]. Certain symptoms of hypothyroidism (carpal tunnel syndrome (*p* = 0.034), having dry or coarse skin (*p* = 0.008), feeling weak (*p* = 0.027)) and weight gain (*p* < 0.01)) were significantly associated with below-median QoL.

Older age was inconsistently associated with poor QoL. In Sawicka-Gutaj et al.’s (2018) open-label study of QoL and an impaired sex life in patients with autoimmune hypothyroidism, the impairment was worse in older patients [84]. Djurovic et al.’s (2018) study of LT4-treated patients with Hashimoto thyroiditis found that QoL was more impaired in patients aged over 50 [86], and Recker et al. (2019) observed partial and inconsistent impairments in ThyPRO and SF-36 in both older patients (aged 60–75) and younger patients (under 40) with SCH before and after treatment [89]. However, a post-hoc analysis of Djurovic et al.’s data suggested that poor QoL was related to weight gain [73].

Other factors that may influence QoL in hypothyroidism include endurance exercise [62], dietary intervention (a diet with green vegetables, beef, whole milk, and butter) [64], higher level of antibodies against thyroid peroxidase [86], the occurrence of severe adverse drug reactions [77], tablet vs. liquid LT4 formulations [44,80], morning vs. evening LT4 administration [58,85], male gender, the duration of hypothyroidism, frequent visits to the general practitioner before diagnosis, negative experiences with LT4, high expectations of LT4, and the provision of support by the general practitioner [104]. However, these factors have not been extensively characterized, and their specificity and selectivity remain to be determined.

#### 3.5.4. Summary

None of the reviewed physiological, genetic, clinical, demographic, or behavioral factors were unambiguously and strongly associated with poor or good QoL. However, some reports suggested that QoL might be influenced by weight gain, the presence of anti-thyroid peroxidase antibodies, physical exercise, marital status, pain levels, employment status, and diet; the exact nature of the relationship between these factors and QoL in hypothyroidism requires further investigation. The diversity of potential influencing factors illustrates the complexity of the relationships between hormone levels, symptoms of hypothyroidism, and QoL. We do not feel that there is a particular age cut-off (65 or otherwise) for effective LT4 replacement therapy. However, older age is always a factor to be taken into account because it is [inconsistently] associated with poor QoL. It is possible again that in older adults with comorbidities, today’s QoL instruments are not always sensitive enough to measure the effects of changes in THRT.

## 4. Discussion

Our review of the literature showed that some aspects of QoL in patients with hypothyroidism have been widely studied over the last 20 years. However, a number of important questions have yet to be addressed. To the best of our knowledge, QoL in patients with benign thyroid disorders was last reviewed by Watt et al. in 2006 [105]. The researchers concluded that “*impairments … are also frequent in the long term*”, i.e., during treatment of hypothyroidism. However, the body of literature on overt hypothyroidism has grown considerably since 2006.

Unsurprisingly, most of the studies reviewed here were performed in Western Europe and the USA. Some European countries were particularly well represented (e.g., The Netherlands, and Denmark), whereas others were notably absent (e.g., France). Data from Asia, Africa, and South America (with the notable exception of Brazil) were lacking or scarce (see below). We noted a wide range of cross-sectional and longitudinal study designs, with a relatively low proportion of double-blind, placebo-controlled RCTs (accounting for around a quarter of the studies reviewed). Many of the randomized studies were prompted by the advent of LT4 + LT3 combination treatment in the early 2000s. There was a notable absence of large, multicenter trials. One of the largest studies was based on a network of 150 general practitioners [71].

Generic and thyroid-specific QoL instruments were applied concomitantly in only 8 of the 58 publications. It should be noted that by virtue of their design, thyroid-specific instruments incorporate both disease symptom ratings and classical QoL domains. This may be problematic because the relationship between symptoms, QoL, and treatment satisfaction is complex. For example, Karmisholt et al.’s (2019) open-label longitudinal study of LT4-treated patients with SCH found that the threshold for a true change in the SF-36 score (20%) was much lower than that for a change in a mood-related symptom score (140%) [87].

A key issue in QoL research is what constitutes “good QoL” in a given population [16,18,19]. This issue is addressed in one of two ways, i.e., by consisting of a local group of euthyroid controls (from the general population or from patients with diseases other than hypothyroidism (e.g., [68,69,72,101]) or by referring to normative data previously collected in the general population [13,14,66]. For example, Wekking et al. (2005) compared their QoL data patients with primary hypothyroidism with normative data from a Dutch general population sample but suggested that a comparison with a control group would have been preferable [66]. The definition of “good” QoL is less of an issue in double-blind, placebo-controlled RCTs that focused on changes over time with treatment or in active treatment vs. placebo groups.

With a few exceptions, most of the reviewed studies included broad populations (with regard to age and the diagnosis or etiology of hypothyroidism) and did not check for the presence of “LT4 nonresponders”. The bulk data suggest that overall QoL is good in LT4-treated patients with hypothyroidism-even when symptoms are present (or persist)-but do not explain why a few percent of biochemically corrected patients complain of poor QoL. A few studies (such as those by Mooijaart et al. [88] and Recker et al. [89]) focused on older patients with SCH, in whom the definition of hypothyroidism differs [106]; we suggest that the assessment of other well-defined patient populations might facilitate the identification of risk factors for poor QoL.

Although we formally excluded studies of LT4-treated patients with differentiated thyroid carcinoma, it is clear from the literature published before the introduction of recombinant TSH that the severe (albeit transient) hypothyroidism following thyroidectomy plus radioiodine ablation therapy is associated with very poor QoL [107]; this may serve as a marker of the “worse possible” QoL experienced in the absence of treatment. However, the relationship between less severe (treated) hypothyroidism (and above all SCH) and poor QoL is less clear. Firstly, the literature data are contradictory with regard to the relationship between the recommended target range for serum TSH (0.4 to 4.0 mU/L) and QoL. On one hand, Walsh et al. [52] and Samuels et al. [81] did not observe differences in QoL between subgroups of treated patients with low normal, high-normal, and/or mildly elevated TSH levels. On the other hand, Dos Santos Vigario et al. [70], Samuels et al. [55], and Mithal et al. [71] concluded that “undertreatment” and “overtreatment (in terms of LT4 dosing) were correlated with significantly worse aspects of QoL.

The randomized studies of LT4 + LT3 combination treatment failed to identify marked differences in QoL after a switch from LT4 alone. During combination treatment (with two types of pill to be taken, etc.), most of the investigators felt that the potential benefits for QoL did not provide a clear clinical rationale in favor of LT4 + LT3. However, the frequent patient preference for LT4 + LT3 (for as-yet unknown reasons) [51] reinforces Wiersinga’s suggestion that robust clinical trials in selected patient subpopulations are essential for making progress in this field [99].

In randomized studies of patients with SCH, THRT did not greatly improve QoL, and QoL was not correlated with thyroid hormone levels–suggesting that residual, treatment-refractory, poor QoL is not solely related to biochemical parameters. This is in line with Feller et al.’s review of SCH [108]. However, the large double-blind, placebo-controlled RCTs in SCH (like IEMO and TRUST) assessed populations of elderly adults [65,100]. This age group does not correspond to the middle-aged adults with SCH who tend to complain of poor QoL–raising the question of whether studies of other patient groups could provide more information on QoL in SCH. In this respect, THRT in SCH might be therefore primarily preventive in nature (to stop or slow progression to overt or clinical hypothyroidism), with the goal of reducing the risk of certain (mainly cardiovascular) complications. However, the absence of an obvious, immediate benefit on QoL might be a crucial argument against treating SCH with LT4.

In most of the studies reviewed here, thyroid function parameters, symptom scores, and hormone concentrations were not correlated with QoL-except in patients with profound hypothyroidism. A few studies identified prognostic factors for QoL. Sowinski et al.’s (2016) prospective case-control study found not only that an increase in the LT4 dose level was associated with changes in symptom scores and QoL among patients with hypothyroid symptoms and normal TSH levels but also that the circulating FT3 concentration was inversely and significantly associated with several domain scores in the ThyPRO (where a lower score indicates better QoL) [74]. A role for T3 is also suggested by trials in which combination therapy was associated with better QoL [56,76,82,83]. Djurovic et al. found that the total SF-36 score was negatively correlated with anti-thyroid peroxidase antibody levels in LT4-treated patients with Hashimoto thyroiditis [86]. It has been suggested that autoimmune disease per se might be associated with poor QoL in hypothyroidism [102]. A few other studies mentioned weight gain, diet, physical exercise, and certain symptoms of hypothyroidism (carpal tunnel syndrome, dry skin, and a feeling of weakness) [62,90]. It is widely accepted that mood, memory, and cognition are affected in hypothyroidism [67,109]. Samuels et al. found that even SCH was associated with specific decrements in working memory and cognitive function [54]- although this relationship is subject to debate in the literature [110]. Given that the standard recall period for the SF-36 is four weeks, one can hypothesize that memory and cognitive impairments may be a confounding factor in some studies. Interestingly, Rezaei et al. recently reported that cognitive-behavioral therapy can improve some aspects of QoL (including emotional health problems, energy, and general health) in patients with hypothyroidism [63]. Lastly, the time of day at which LT4 was taken (morning vs. evening) did not appear to change QoL in adults [58] or children [85].

The present study had a number of limitations. Firstly, only the PubMed database was searched. Secondly, only publications in English were screened. These possible sources of bias might explain why few of the selected publications came from the Asia-Pacific region. Thirdly, the results obtained with a given QoL questionnaire in one study cannot be directly compared with those obtained in a different study. The study also had some strengths: it notably constituted the first extensive review of QoL in hypothyroidism and identified some important topics for further research.

## 5. Conclusions

Our review of the literature indicated that THRT restores QoL to often near-normal levels within 3 to 6 months. However, discordant results were found-especially in patients with SCH. Combination treatment with LT4 + LT3 does not appear to lead to better QoL. Very few prognostic factors for QoL have been identified but these may include weight gain, physical activity, autoimmunity, and diet, and may not necessarily be related to thyroid endocrine status. However, the factors that influence QoL in hypothyroidism—especially in “treatment-resistant” poor QoL observed in symptomatic SCH—require further characterization. Perspectives for further research on QoL in hypothyroidism might include (i) concomitantly evaluations with both a thyroid-specific questionnaire and a generic HR-QoL questionnaire, (ii) studies of QoL in nonelderly populations with SCH, (iii) the choice of control patient groups as a guide to “normal” QoL, (iv) investigations of why QoL subdomain scores sometimes change in opposite directions (e.g., an improvement in physical QoL and a worsening in mental QoL) and (v) investigation of the relationships between QoL questionnaires, symptoms, and other patient-reported outcomes (e.g., the apparent patient preference for LT4 + LT3 combination treatment [51]).

## Figures and Tables

**Table 1:** Summary of the characteristics of quality of life questionnaires used in the reviewed publications.

Abbreviated and Full Name [Reference]	Score Items or Domains	Scoring and Scale
Thyroid-specific health-related QoL instruments		
ThyDQoL, Underactive Thyroid- Dependent Quality of Life Questionnaire [23]	20 items on present QoL, thyroid-dependent QoL, spare time, working life, holidays, family life, social life, closest personal relationship, sex life, physical capability, energy, speed, getting around, household tasks, appearance, weight, bodily discomfort, depression, motivation.	Items are rated on a 7-point scale from excellent to extremely bad or on a 5-point scale from very much better to worse. Weighted domain scores and the average weighted impact score range from −9 (worst QoL) to +3 (best QoL).
ThyPRO, Thyroid-Specific Patient-Reported Outcome Measure [24]	84 self-reported items in 13 scales: Goiter symptoms, Hyperthyroid symptoms, Hypothyroid symptoms, Eye symptoms, Tiredness, Cognitive impairment, Anxiety, Depressivity, Emotional susceptibility, Impaired social life, Impaired daily life, Impaired sex life, Cosmetic complaints.	Each item is rated by the patient on a five-point Likert scale. Some items require the patients to state whether or not a particular condition has been diagnosed by a physician. After transformation, each scale score ranges from 0 (best QoL) to 100 (worst QoL).
HRQL, Hypothyroid- Specific Health-Related Quality of Life Questionnaire [25]	29 items on Weight gain; Feeling colder than others around you; Generally unwell; Needing nap during the day; Slower physically; No energy to get through the day; Loss of interest in hobbies or enjoyable activities; Difficulty remembering things; Dry skin; Brittle nails; Constipation; Need for more sleep; Exhausted; Slower mentally; Lethargic; Depressed; Frustrated; Difficulty concentrating; Muscle weakness; Puffiness of hands; Tired; Less energetic; Sluggish throughout the day; More worn out; Worried; Discouraged; Deterioration of memory	The severity of the discomfort or problem during the preceding month is specified on a 5-point scale ranging from “not at all” (1 point) to “all the time” (5 points). The overall score ranges from 29 (worst QoL) to 145 (best QoL).
ThyTSQ, Underactive Thyroid Treatment Satisfaction Questionnaire [26]	10 items covering satisfaction with current treatment, convenience, and understanding of treatment.	Patients respond to each item by circling a number on a scale from 6 to 0, indicating their degree of satisfaction with that aspect of treatment e.g., from very satisfied to very dissatisfied.
A modified Chronic Thyroid Questionnaire [27].	The original Chronic Thyroid Questionnaire [28] contains up to 104 items grouped into four domains: physical; energy and wellbeing; mood/ emotions; and cognitive functioning. The patient identifies applicable items (potentially ranging from 0 to 104, making intra- and inter-patient comparisons difficult) and rates the corresponding degree of discomfort on a zero-to-five scale, where zero is the most favorable.	Kaminski et al. [27] selected 29 items from the Chronic Thyroid Questionnaire and added four items (palpitation, insomnia, irritability, and anxiety). The 33 items were grouped into three categories: physical complaints (12 items), energy, and general well-being (11 items), and mood and emotions (10 items). Each item is scored on a zero-to-five scale.
Generic health-related QoL instruments		
MOS, Medical Outcomes Study Health Status Questionnaire, core questionnaire [29]	116 items in 9 domains:physical functioning, physical role, bodily pain, general health, vitality, social functioning, emotional role, cognitive functioning scale, mental health index	Each section is scored from 0 (worst QoL) to 100 (best QoL), with reference to the preceding month
MOS SF-20, Medical Outcomes Study Short Form-20 [30]	20 items in 6 domains: physical functioning, role functioning, social functioning, mental health, current health perceptions, pain	Each section is scored from 0 (worst QoL) to 100 (best QoL), with reference to the preceding month.
SF-36, Short Form (36) Health Survey [31]	36 items in 8 domains: vitality, physical functioning, bodily pain, general health perceptions, physical role functioning, emotional role functioning, social role functioning, mental health	36 items scored variously on 2-point (yes/no), 3-point, and (predominantly) 5-point scales, with reference to the preceding month. Each section is scored from 0 (worst QoL) to 100 (best QoL). A physical composite score (PCS) and a mental composite score (MCS) can be calculated from the section scores, using a proprietary algorithm. These are also scored from 0 (worst QoL) to 100 (best QoL).
RAND-36, the RAND 36-item Health Survey [32]	36 items in 8 domains (the same as in the SF-36): vitality, physical functioning, bodily pain, general health perceptions, physical role functioning, emotional role functioning, social role functioning, mental health	Items are scored variously on 2-point (yes/no), 3-point, and (predominantly) 5-point scales, with reference to the preceding month. Compared with the SF-36, the scoring method differs for the general health perceptions and bodily pain sections. Each section is scored from 0 (worst QoL) to 100 (best QoL).
GHQ-12, General Health Questionnaire 12-items [33,34,35].	12 items in 3 psychological domains: Anxiety and insomnia, Social dysfunction, Loss of confidence	The items are rated from 0 to 3. The total score ranges from 0 (best QoL) to 36 (worst QoL). The original GHQ had 60 items. The 12-item version is most frequently used but 28- and 30-item versions are also available [34,35].
PedsQL, Pediatric Quality of Life Inventory [36]	23 items in 4 domains: physical functioning, emotional functioning, social functioning, school functioning	Each item is a score on a Likert scale from 0 to 4. A Psychosocial Health Summary Score and the Physical Health Summary Score can be computed, along with a total score ranging from 0 (worst QoL) to 100 (best QoL).
WHOQoL-Bref, the Abbreviated World Health Organization Quality of Life Instrument [37]	26 items in four broad domains: physical health, psychological health, social relationships, and environment.	Each section is scored from 0 (worst QoL) to 100 (best QoL). An overall score, computed using an algorithm, also ranges from 1 (worst) to 100 (best).
EQ-5D, EuroQol five-dimensional questionnaire [38]	Two components: health state description (in five dimensions: mobility, self-care, usual activities, pain/discomfort, and anxiety/depression) and evaluation (overall health status, on a visual analog scale).	The level of severity for each dimension is rated on three levels (EQ-5D-3L) or five levels (EQ-5D-5L), generating 243 unique health states. These states are converted into an index utility score, ranging from −0.59 (worst QoL) to 1.00 (best QoL). The 20 cm visual analog scale is rated from 0 to 100
EORTC QLQ-C30, European Organization for Research and Treatment Core Quality of Life Questionnaire [39]	30 items with 5 functional scales (physical, role, cognitive, emotional, and social); 3 symptom scales (fatigue, pain, and nausea and vomiting); and a global health and quality-of-life scale. The core questionnaire was developed for use with cancer patients.	Each item is rated on a scale of 1 to 4. The overall score ranges from 30 (best QoL) to 120 (worst QoL).
COOP/WONCA, Primary Care Cooperative Information Project/World Organization of National Colleges, Academies and Academic Associations of General Practitioners/Family Physicians functional status questionnaire [40,41]	Six single-item scales: physical fitness, feelings, daily activities, social activities, change in health, and overall health	Each chart is rated on a 5-point scale ranging from 1 (best functional status) to 5 (worst functional status). The developers do not recommend the use of a summative (total) score.
NHP, Nottingham Health Profile [42]	Part 1. 38 items (yes/no answers) yielding 6 “packages”: physical mobility, pain, energy, sleep, emotional reactions, and social isolation. Part 2 (optional). 7 items (yes/no answers) on occupation, housework, social life, family life, sexual function, hobbies, and holidays.	For each package: 0 (best QoL) to 100 (worst QoL). A total score is obtained by averaging the package score, and so also ranges from 0 (best QoL) to 100 (worst QoL).
KINDL-R [43]	A self-reported or parent-reported questionnaire with 24 items assessing 6 dimensions: physical well-being, emotional wellbeing, self-esteem, family, friends, and everyday functioning	Each item is a score on a five-point Likert scale (1 to 5). The total score is transformed into a value of between 0 (worst QoL) and 100 (best QoL).

**Table 2:** Reviewed randomized studies, by year of publication.

First Author	Publication Year and Journal	Topic	Study Design	Country (Number of Investigating Centers)	Indication/Population	QoL Instrument(s) Scored, and Rank of QoL Outcome *	Difference or Change in QoL
Pollock [46]	BMJ 2001	LT4 vs. placebo in SCH	Double-blind randomized crossover trial	UK (1)	Patients with symptoms of hypothyroidism (*n* = 22)	SF-36 (secondary outcome)	⇔ for LT4 vs. placebo
Clyde [25]	JAMA 2003	LT4 vs. combination therapy LT4 + LT3	Double-blind, randomized trial	USA (not reported)	Primary hypothyroidism (mostly due to autoimmune disease) (*n* = 44 (22 + 22))	HRQL (primary outcome)	⇔ for LT4 + LT3 vs. LT4
Sawka [47]	J Clin Endocrinol Metab 2003	LT4 vs. combination therapy LT4 + LT3	Double-blind randomized clinical trial	USA (1)	Primary hypothyroidism treated for at least 6 months (*n* = 40 (20 + 20))	MOS (secondary outcome)	⇔ for LT4 vs. LT4 + LT3
Walsh [48]	J Clin Endocrinol Metab 2003	LT4 vs. combination therapy LT4 + LT3	Double-blind randomized crossover trial	Australia (1 referral center)	Primary hypothyroidism with stable LT4 treatment (*n* = 110 (101 completers))	SF-36 (secondary outcome)	⇔ for LT4 vs. LT4 + LT3
Roos [49]	Arch Intern Med 2005	LT4 dose levels Low-dose vs. normal-dose LT4 in cardiac asymptomatic hypothyroidism	Double-blind, randomized clinical trial	The Netherlands (1)	Newly diagnosed, untreated primary autoimmune hypothyroidism (*n* = 50 (25 on low starting dose))	RAND-36 (secondary outcome)	⇔ for low LT4 starting dose vs. full LT4 starting dose
Escobar-Morreale [50]	Ann Intern Med 2005	LT4 vs. combination therapy LT4 + LT3	Double-blind, randomized crossover trial.	Spain (1)	Women with overt primary hypothyroidism (*n* = 26)	SF-36 (secondary outcome)	⇔ for LT4 vs. LT4 + LT3
Appelhof [51]	J Clin Endocrinol Metab 2005	LT4 vs. combination therapy LT4 + LT3	Double-blind randomized clinical trial	The Netherlands (1)	LT4 for primary autoimmune hypothyroidism for at least 6 months (*n*= 130 completers (44 + 41 + 45))	RAND-36 (secondary outcome)	⇔ for LT4 vs. LT4 + LT3
Walsh [52]	J Clin Endocrinol Metab 2006	LT4 dose levels	Double-blind, randomized crossover trial	Australia (1)	Primary hypothyroidism (autoimmune hypothyroidism or Graves or benign thyroid cancer) (*n* = 50 completers)	SF-36 (secondary outcome)	⇔ between LT4 dose levels
Razvi [53]	J Clin Endocrinol Metab 2007	Subclinical hypothyroidism	Double-blind, randomized crossover study of LT4 vs. placebo.	UK (27 general practices)	Stable SCH (*n* = 100, 99 completers)	ThyDQoL (secondary outcome)	⇔ for LT4 vs. placebo
Samuels [54]	J Clin Endocrinol Metab 2007	SCH and QoL	Double-blind, randomized crossover study	USA (1)	13 with autoimmune hypothyroidism and six treated for Graves’ disease (*n* = 19)	SF-36 (secondary outcome)	⇔ for usual vs. lower LT4 dose
Samuels [55]	J Clin Endocrinol Metab 2008	Usual LT4 dose vs. high-dose LT4	Double-blind, randomized, cross-over study	USA (not reported)	Adult-onset primary hypothyroidism (autoimmune or Graves’ disease) (*n* = 33)	SF-36 (secondary outcome)	↓ for high-dose LT4 vs. normal-dose LT4
Nygaard [56]	Eur J Endocrinol 2009	switch from LT4 to LT4 + LT3	Double-blind, randomized cross-over study	Denmark (3)	Overt, spontaneous hypothyroid subjects on LT4 for at least 6 months (*n* = 59)	SF-36 (primary outcome)	↓ with LT4 vs. LT4 + LT3
Panicker [57]	J Clin Endocrinol Metab 2009	QoL in LT4-treated patients	Cross-sectional study of a randomized trial population	UK (28)	Patients taking at least 100 µg/day LT4 and with DNA for genotyping (*n* = 552)	GHQ-12 (primary outcome)	↑ in response to LT4 + LT3 vs. LT4 (but only for some patients with a *DIO2* polymorphism)
Bolk [58]	Arch Intern Med 2010	Time of administration: evening or morning	Double-blind, randomized crossover trial	The Netherlands (1)	Primary hypothyroidism (*n* = 90 (47 + 43))	SF-36 (secondary outcome)	⇔ for morning vs. evening
Reuters [59]	Arq Bras Endocrinol Metabol 2012	SCH	Double-blind, randomized clinical trial	Brazil (1)	SCH (*n* = 35 (25 finished the study))	SF-36 (secondary outcome)	↑ with LT4 vs. placebo
Hoang [60]	J Clin Endocrinol Metab 2013	Switch from LT4 to DTE	Double-blind randomized crossover study	USA (1)	Primary hypothyroidism and a stable dose of LT4 for at least 6 months (*n* = 70)	GHQ-12 (one of several primary outcomes)	⇔ for DTE vs. LT4
Kaminski [27]	Arch Endocrinol Metab 2016	Switch from LT4 to LT4 + LT3– primary hypothyroidism	Double-blind, randomized crossover study.	Brazil (1)	Hypothyroidism, stable doses of LT4 during the previous six months (*n* = 32)	HRQoL questionnaire (a modified version of the Chronic Thyroid Questionnaire) (secondary outcome ?)	⇔ for LT4 vs. LT4 + LT3
Stott [61]	N Engl J Med 2017	QoL in older patients with SCH	Double-blind placebo- controlled randomized trial	The Netherlands, UK, Eire, Switzerland (not reported)	Adults aged 65 or over with SCH (*n* = 638 (318 + 320))	ThyPRO and EQ-5D (primary outcome)	⇔ for LT4 vs. placebo
Werneck study 2 [62]	Arch Endocrinol Metab 2018	SCH and exercise	Randomized controlled trial	Brazil (1)	SCH (*n* = 20 (10 + 10))	SF-36 (primary outcome)	↑ with aerobic exercise training
Rezaei [63]	Thyroid Res 2020	QoL in LT4-treated patients: effect of cognitive behavioral therapy	Open-label randomized controlled trial	Iran (1)	Women of child-bearing age with hypothyroidism (*n* = 86 (43 + 43))	SF-36 (primary outcome)	↑ with cognitive behavioral therapy
van der Gaag [64]	Int J Environ Res Public Health 2020	Effect of a dietary intervention (green vegetables, beef, whole milk and butter) on QoL in SCH	Open-label randomized controlled trial	The Netherlands (2)	Children aged 1–12 with a pediatrician- confirmed diagnosis of SCH (*n* = 61 (29 + 32))	PedsQL (secondary outcome)	↑ with diet improvement vs. controls
de Montmollin [65]	Ann Intern Med 2020	QoL in older patients with SCH	Double-blind placebo- controlled randomized trial	The Netherlands, UK, Eire, Switzerland (not reported)	Adults aged 65 or over with SCH (*n* = 638 (66 + 66 + 252 + 254))	ThyPRO (primary outcome) and EQ-5D (secondary outcome)	⇔ for LT4 vs. placebo

LT4: levothyroxine; SCH: subclinical hypothyroidism; LT3: L-tri-iodothyronine; HRQL: Hypothyroid-specific health-related quality of life questionnaire; T3: tri-iodothyronine; T4: thyroxine; SF-36: Short Form (36) Health Survey; GHQ-28: 28-item General Health Questionnaire; QoL: quality of life; RAND: RAND Corporation; PCS: physical composite score; ThyDQoL: Thyroid-Dependent Quality of Life Questionnaire; MCS: mental composite score; FT3: free tri-iodothyronine; FT4: free thyroxine; DIO: deiodinase; DTE: dried thyroid extract; ThyPRO: Thyroid-Specific Patient-Reported Outcome Measure; PedsQL, Pediatric Quality of Life Inventory. * We considered that QoL was the study’s primary outcome when it was stated as such in the publication or, in the absence of such a statement, when it was the first-mentioned outcome in a list or was most the comprehensively reported outcome. ↑ significantly better QoL or a significant improvement in QoL; ⇔ no significant difference or change in QoL; ↓ significantly worse QoL or a significant decrease in QoL.

**Table 3:** Reviewed nonrandomized studies, by year of publication.

First Author	Publication Year and Journal	Topic	Study Design	Country (Number of Investigating Centers)	Indication/Population	Qol Instrument(S) Scored, and Rank of Qol Outcome *	Difference or Change in Qol
Wekking [66]	Eur J Endocrinol 2005	QoL in treated hypothyroidism	Cross-sectional study	The Netherlands (13 general practices, 1 referral center)	Primary hypothyroidism during LT4 treatment (*n* = 141)	RAND-36 (secondary outcome)	↓ in LT4-treated patients vs. general population
Gulseren [67]	Arch Med Res 2006	QoL in treated hypothyroidism	Cross-sectional study vs. controls	Turkey (1)	Overt hypothyroidism and SCH (also overt hyperthyroidism and subclinical hyperthyroidism) (*n* = 76 (43 SCH, 33 overt))	SF-36 (secondary outcome)	↑ on treatment
Samuels [68]	Thyroid 2007	QoL in LT4-treated patients	Cross-sectional comparative study	USA (1)	Primary hypothyroidism (autoimmune hypothyroidism or Graves’ disease or benign thyroid cancer) (*n* = 34)	SF-36 (secondary outcome)	↓ for lower LT4 dose vs. usual LT4 dose
McMillan [23]	Value Health 2008	QoL in LT4-treated overt or SCH	Study of psychometric properties of ThyDQoL	UK (1)	Overt hypothyroidism and SCH (*n* = 110, (103 treated))	ThyDQoL (primary outcome)	↓ in LT4-treated patients
Quinque [69]	Health Qual Life Outcomes 2013	Adequately treated hypothyroidism	Scale validation study and then a longitudinal study vs. healthy controls	Germany (1)	Diagnosed hypothyroidism (*n* = 18).	ThyDQoL (primary outcome)	⇔ for patients vs. controls
dos Santos Vigario [70]	Endocrine 2013	TSH levels (“overtreated” etc.)	Cross-sectional study	Brazil (4)	Consecutive primary hypothyroidism patients on LT4 replacement (*n* = 33)	SF-36 (primary outcome)	↓ in “under-treated” patients (TSH >4.0 mIU/L, i.e., insufficient LT4)
Mithal [71]	Indian J Endocrinol Metab 2014	LT4 dosing after treatment initiation	Cross-sectional study	India (150 physicians from 10 cities)	Primary hypothyroid patients with abnormal thyroid function despite being prescribed LT4 for at least 2 months (*n* = 1950)	SF-36 (secondary outcome)	↓ in “under-treated” patients (i.e., insufficient LT4)
Samuels [72]	J Clin Endocrinol Metab 2014	“Over-treated” hypothyroidism	Cross-sectional study	USA (1)	Women receiving chronic TSH-suppressive LT4 doses (low-risk thyroid cancer or overtreatment of hypothyroidism), chronic replacement LT4 doses, or no LT4 (*n* = 59)	SF-36 (primary outcome)	↓ for LT4-treated patients vs. healthy controls
Kelderman-Bolk [73]	Eur J Endocrinol 2015	Factors associated with QoL (weight gain)	Baseline survey before a cross-over trial	The Netherlands (1)	Treated primary hypothyroidism (*n* = 90)	SF-36 (primary outcome)	↓ for LT4-treated patients vs. healthy controls
Winther [13]	PLoS One 2016	QoL in newly treated patients	Prospective cohort study	Denmark (2)	Hypothyroidism due to autoimmune thyroiditis (*n* = 78)	SF-36 and ThyPRO (primary outcomes)	↑ in LT4-treated patients
Sowinski [74]	Pol Arch Med Wewn 2016	Relationship between QoL and the dose level of LT4	Prospective case-control study	Poland (1)	Patients with hypothyroidism (Hashimoto thyroiditis, thyroidectomy, radioiodine) before and after a dose increase in LT4 (*n* = 33)	ThyPRO (primary outcome)	↑ in LT4-treated patients
Samuels [75]	Thyroid 2016	Relationship between TSH levels and QoL	Cross-sectional study	USA (1)	Healthy hypothyroid subjects receiving chronic replacement therapy with levothyroxine (LT4) (*n* = 132, (85 low-normal TSH + 47 high-normal TSH))	SF-36 (primary outcome)	⇔ for different TSH levels
Jonklaas and Burman [76]	Thyroid 2016	Switch from LT4 to LT3	Open-label, single-arm study	USA (1)	Hypothyroidism of any etiology, no significant medical problems, LT4 dose of 75 µg (*n* = 18)	SF-36 (secondary outcome)	↑ (slight) for LT3 monotherapy
Rolfes [77]	Drug Saf 2016	Changes in QoL after an ADR	Cross-sectional survey	The Netherlands (1)	Patients who experienced an ADR after a packaging change and who reported this to a pharmacovigilance center (*n* = 1167)	COOP/WONCA questionnaire (primary outcome)	↓ after an ADR due to a packaging change
Young Cho [78]	Endocrine 2017	Polymorphisms in iodothyronine deiodinase	Open-label cross-sectional study	Korea (1)	Chronic autoimmune thyroiditis and thyroid cancer (*n* = 136 and *n* = 60)	SF-36 and ThyPRO (primary outcomes)	↓ for DIO1 variants ⇔ for DIO2 and 3 variants
Wouters [79]	Thyroid 2017	Polymorphisms in iodothyronine deiodinase (DIO)	Cross-sectional study	The Netherlands (regional population-based cohort)	Patients taking LT4 (*n* = 364 LT4 users (146 Thr/Thr, 140 Thr/Ala, 35 Ala/Ala))	RAND 36-Item Health Survey (primary outcome)	⇔ in LT4-treated patients ⇔ no significant effect of the D2-Thr92Ala polymorphism
Lombardi [80]	Endocrine 2017	Tablet vs. liquid formulations of LT4	Open-label longitudinal study	Italy (1)	Total thyroidectomy (*n* = 155 (77 liquid, 78 tablet))	GHQ-12 (secondary outcome)	↑ for a liquid formulation vs. tablet
Samuels [81]	J Clin Endocrinol Metab 2018	Targeting TSH levels	Open-label longitudinal study	USA (1)	Hypothyroidism (*n* = 138)	ThyDQoL, SF-36 (primary outcomes)	⇔ for different TSH levels
Werneck study 1 [62]	Arch Endocrinol Metab 2018	SCH and exercise	Open-label cross-sectional study SCH vs. euthyroid	Brazil (1)	SCH (*n* = 22)	SF-36 (primary outcome)	⇔ for SCH vs. euthyroid individuals
Michaelsson [82]	Eur Thyroid J 2018	LT4 vs. combination therapy LT4 + LT3 in SCH	Open-label longitudinal cohort study	Denmark (1)	SCH (*n* = 23)	ThyPRO (primary outcome)	↑ for a switch to LT4 + LT3 from LT4
Tariq [83]	South Med J 2018	Switch from LT4 to LT4 + LT3 or DTE	Observational retrospective study	USA (1)	Hypothyroidism on “optimal” LT4 only (*n* = 100 (40 switched to LT4/LT3, 60 switched to DTE))	MOS Short Form-20 (primary outcome)	↑ for a switch to LT4 + LT3 from LT4
Sawicka-Gutaj study 1 [84]	Thyroid 2018	Impaired sex life	Open-label cross-sectional study	Denmark (2)	Autoimmune hypothyroidism (one subgroup) (*n* = 189)	SF-36 and ThyPRO (primary outcomes)	↓ before LT4 treatment
Sawicka-Gutaj study 2 [84]	Thyroid 2018	Impaired sex life	Open-label longitudinal study	Denmark (2)	Autoimmune hypothyroidism (one subgroup) (*n* = 86)	SF-36 and ThyPRO (primary outcomes)	↑ with LT4 treatment
Guglielmi [44]	Endocr Metab Immune Disord Drug Targets 2018	Tablet vs. liquid formulations of LT4	Open-label longitudinal study	Italy (2)	Hypothyroidism with stable TSH levels on LT4 (*n* = 102)	ThyTSQ (primary outcome)	↑ for liquid LT4 taken at breakfast
Akın [85]	J Pediatr Endocrinol Metab 2018	Regimen timing–pediatric hypothyroidism	Open-label cross-sectional study of bedtime vs. evening LT4 regimens	Turkey (not reported)	Acquired hypothyroidism (*n* = 163)	PedsQL (secondary outcome)	⇔ for morning vs. bedtime dosing
Djurovic [86]	Endocrine 2018	Factors associated with QoL (Hashimoto thyroiditis)	Open-label cross-sectional study vs. controls	Serbia (1)	Hashimoto thyroiditis (*n* = 130 (59 20–49 years old + 71 >50 years old))	SF-36 (primary outcome)	↓ in LT4-treated patients vs. controls
Karmisholt [87]	Eur Thyroid J 2019	SCH	Open-label longitudinal cohort study	Denmark (1)	SCH (*n* = 15)	SF-36 (primary outcome)	⇔ vs. TSH levels
Mooijaart [88]	JAMA 2019	SCH–older patients	Cross-sectional study	Several European countries. 1st trial: The Netherlands and Switzerland. 2nd trial: Netherlands, Switzerland, Ireland, and the UK (not reported)	SCH (*n* = 93)	ThyPRO (primary outcome), EuroQol-5D, EuroQol VAS (secondary outcomes)	⇔ for LT4-treated patients
Recker [89]	Horm Metab Res 2019	SCH–older patients	Open-label longitudinal cohort study	Germany (6 endocrine practices)	Newly diagnosed, untreated, overt endogenous hypothyroidism or SCH (*n* = 28 (11 > 60 years + 17 < 40 years ))	ThyPRO and SF-36 (primary outcomes)	↑ for LT4-treated patients
Tan [90]	Fam Pract 2019	Factors associated with QoL	Cross-sectional survey	Singapore (1)	Hypothyroidism (*n* = 229)	EuroQol-5D-5L (primary outcome)	↓ for patients with symptoms and comorbidities
Hirtz [91]	Front Endocrinol (Lausanne) 2020	QoL in children and adolescents with thyroid disease	Cross-sectional survey	Germany (not reported)	Subclinical and overt hypothyroidism, Subclinical and overt hyperthyroidism, Hashimoto’s thyroiditis (*n* = 351; 331 (subclinical) + 20 (overt)	KINDL-R (primary outcome)	⇔ for the various disease groups
Ur Rehman [92]	J Ayub Med Coll Abbottabad 2020	QoL in treated hypothyroidism	Cross-sectional survey	Pakistan (1)	Patients aged 18–60 with confirmed hypothyroidism (*n* = 52)	ThyPRO (primary outcome)	↑ upon LT4 treatment
Moron-Diaz [93]	Endocrine 2020	QoL in LT4-treated patients	Cross-sectional survey	Spain (1)	Patients with adequately treated primary hypothyroidism of any cause (*n* = 218)	ThyPRO (primary outcome)	↑ for lower TSH levels

LT4: levothyroxine; SCH: subclinical hypothyroidism; LT3: L-tri-iodothyronine; HRQL: Hypothyroid-specific health-related quality of life questionnaire; SF-36: Short Form (36) Health Survey; QoL: quality of life; RAND: RAND Corporation; PCS: physical composite score; ThyDQoL: Thyroid-Dependent Quality of Life Questionnaire; MCS: mental composite score; FT3: free tri-iodothyronine; FT4: free thyroxine; DIO: deiodinase; TSH: thyroid-stimulating hormone; TPO-Ab: antibodies against thyroid peroxidase; DTE: dried thyroid extract; ThyPRO: Thyroid-Specific Patient-Reported Outcome Measure; ADR: adverse drug reaction; TG-Ab: antibodies against thyroglobulin; PedsQL, Pediatric Quality of Life Inventory; ThyTSQ: Underactive Thyroid Treatment Satisfaction Questionnaire. * We considered that QoL was the study’s primary outcome when it was stated as such in the publication or, in the absence of such a statement, when it was the first-mentioned outcome in a list or was most the comprehensively reported outcome. ↑ significantly better QoL or a significant improvement in QoL; ⇔ no significant difference or change in QoL; ↓ significantly worse QoL or a significant decrease in QoL.

## Data Availability

Not applicable.

## References

[1] Chaker L., Bianco A.C., Jonklaas J., Peeters R.P. (2017). Hypothyroidism. Lancet.

[2] Garmendia Madariaga A., Santos Palacios S., Guillen-Grima F., Galofre J.C. (2014). The incidence and prevalence of thyroid dysfunction in Europe: A meta-analysis. J. Clin. Endocrinol. Metab..

[3] Aoki Y., Belin R.M., Clickner R., Jeffries R., Phillips L., Mahaffey K.R. (2007). Serum TSH and total T4 in the United States population and their association with participant characteristics: National Health and Nutrition Examination Survey (NHANES 1999–2002). Thyroid.

[4] Biondi B., Cappola A.R., Cooper D.S. (2019). Subclinical Hypothyroidism: A Review. JAMA.

[5] Cooper D.S. (2001). Clinical practice. Subclinical hypothyroidism. N. Engl. J. Med..

[6] Gosi S.K.Y., Garla V.V. (2020). Subclinical Hypothyroidism.

[7] Hennessey J.V. (2017). The emergence of levothyroxine as a treatment for hypothyroidism. Endocrine.

[8] Jonklaas J., Bianco A.C., Bauer A.J., Burman K.D., Cappola A.R., Celi F.S., Cooper D.S., Kim B.W., Peeters R.P., Rosenthal M.S. (2014). Guidelines for the treatment of hypothyroidism: Prepared by the american thyroid association task force on thyroid hormone replacement. Thyroid.

[9] Mateo R.C.I., Hennessey J.V. (2019). Thyroxine and treatment of hypothyroidism: Seven decades of experience. Endocrine.

[10] Biondi B., Cooper D.S. (2019). Thyroid hormone therapy for hypothyroidism. Endocrine.

[11] Villar H.C., Saconato H., Valente O., Atallah A.N. (2007). Thyroid hormone replacement for subclinical hypothyroidism. Cochrane Database Syst. Rev..

[12] Sheehan M.T. (2016). Biochemical Testing of the Thyroid: TSH is the Best and, Oftentimes, Only Test Needed—A Review for Primary Care. Clin. Med. Res..

[13] Winther K.H., Cramon P., Watt T., Bjorner J.B., Ekholm O., Feldt-Rasmussen U., Groenvold M., Rasmussen A.K., Hegedus L., Bonnema S.J. (2016). Disease-Specific as Well as Generic Quality of Life Is Widely Impacted in Autoimmune Hypothyroidism and Improves during the First Six Months of Levothyroxine Therapy. PLoS ONE.

[14] Bianchi G.P., Zaccheroni V., Solaroli E., Vescini F., Cerutti R., Zoli M., Marchesini G. (2004). Health-related quality of life in patients with thyroid disorders. Qual. Life Res..

[15] Wiersinga W.M., Duntas L., Fadeyev V., Nygaard B., Vanderpump M.P. (2012). 2012 ETA Guidelines: The Use of L-T4 + L-T3 in the Treatment of Hypothyroidism. Eur. Thyroid J..

[16] Calman K.C. (1984). Quality of life in cancer patients—An hypothesis. J. Med. Ethics.

[17] Diener E., Oishi S., Lucas R.E. (2003). Personality, culture, and subjective well-being: Emotional and cognitive evaluations of life. Annu. Rev. Psychol..

[18] Campbell A. (1976). Subjective measures of well-being. Am. Psychol..

[19] Leplege A., Hunt S. (1997). The problem of quality of life in medicine. JAMA.

[20] Grani G., Ramundo V., Verrienti A., Sponziello M., Durante C. (2019). Thyroid hormone therapy in differentiated thyroid cancer. Endocrine.

[21] Carlson L.E., Waller A., Groff S.L., Giese-Davis J., Bultz B.D. (2013). What goes up does not always come down: Patterns of distress, physical and psychosocial morbidity in people with cancer over a one year period. Psychooncology.

[22] Barbus E., Pestean C., Larg M.I., Piciu D. (2017). Quality of life in thyroid cancer patients: A literature review. Clujul Med..

[23] McMillan C., Bradley C., Razvi S., Weaver J. (2008). Evaluation of new measures of the impact of hypothyroidism on quality of life and symptoms: The ThyDQoL and ThySRQ. Value Health.

[24] Watt T., Hegedus L., Rasmussen A.K., Groenvold M., Bonnema S.J., Bjorner J.B., Feldt-Rasmussen U. (2007). Which domains of thyroid-related quality of life are most relevant? Patients and clinicians provide complementary perspectives. Thyroid.

[25] Clyde P.W., Harari A.E., Getka E.J., Shakir K.M. (2003). Combined levothyroxine plus liothyronine compared with levothyroxine alone in primary hypothyroidism: A randomized controlled trial. JAMA.

[26] McMillan C.V., Bradley C., Woodcock A., Razvi S., Weaver J.U. (2004). Design of new questionnaires to measure quality of life and treatment satisfaction in hypothyroidism. Thyroid.

[27] Kaminski J., Miasaki F.Y., Paz-Filho G., Graf H., Carvalho G.A. (2016). Treatment of hypothyroidism with levothyroxine plus liothyronine: A randomized, double-blind, crossover study. Arch. Endocrinol. Metab..

[28] Jaeschke R., Guyatt G., Cook D., Harper S., Gerstein H.C. (1994). Spectrum of quality of life impairment in hypothyroidism. Qual. Life Res..

[29] Brook R.H., Davies-Avery A., Greenfield S., Harris L.J., Lelah T., Solomon N.E., Ware J.E. (1977). Assessing the quality of medical care using outcome measures: An overview of the method. Med. Care.

[30] Stewart A.L., Hays R.D., Ware J.E. (1988). The MOS short-form general health survey. Reliability and validity in a patient population. Med. Care.

[31] Ware J.E., Sherbourne C.D. (1992). The MOS 36-item short-form health survey (SF-36). I. Conceptual framework and item selection. Med. Care.

[32] Hays R.D., Sherbourne C.D., Mazel R.M. (1993). The RAND 36-Item Health Survey 1.0. Health Econ..

[33] Goldberg D.P., Blackwell B. (1970). Psychiatric illness in general practice. A detailed study using a new method of case identification. Br. Med. J..

[34] Williams P., Goldberg D.P. (1988). A User’s Guide to the General Health Questionnaire.

[35] Goldberg D.P., Hillier V.F. (1979). A scaled version of the General Health Questionnaire. Psychol. Med..

[36] Varni J.W., Seid M., Kurtin P.S. (2001). PedsQL 4.0: Reliability and validity of the Pediatric Quality of Life Inventory version 4.0 generic core scales in healthy and patient populations. Med. Care.

[37] Skevington S.M., Lotfy M., O’Connell K.A., WHOQOL Group (2004). The World Health Organization’s WHOQOL-BREF quality of life assessment: Psychometric properties and results of the international field trial. A report from the WHOQOL group. Qual. Life Res..

[38] EuroQol Group (1990). EuroQol—A new facility for the measurement of health-related quality of life. Health Policy.

[39] Aaronson N.K., Bullinger M., Ahmedzai S. (1988). A modular approach to quality-of-life assessment in cancer clinical trials. Recent Results Cancer Res..

[40] Kinnersley P., Peters T., Stott N. (1994). Measuring functional health status in primary care using the COOP-WONCA charts: Acceptability, range of scores, construct validity, reliability and sensitivity to change. Br. J. Gen. Pract..

[41] Nelson E., Wasson J., Kirk J., Keller A., Clark D., Dietrich A., Stewart A., Zubkoff M. (1987). Assessment of function in routine clinical practice: Description of the COOP Chart method and preliminary findings. J. Chronic Dis..

[42] Hunt S.M., McKenna S.P., McEwen J., Backett E.M., Williams J., Papp E. (1980). A quantitative approach to perceived health status: A validation study. J. Epidemiol. Community Health.

[43] Ravens-Sieberer U., Erhart M., Wille N., Bullinger M., BELLA study group (2008). Health-related quality of life in children and adolescents in Germany: Results of the BELLA study. Eur. Child Adolesc. Psychiatry.

[44] Guglielmi R., Grimaldi F., Negro R., Frasoldati A., Misischi I., Graziano F., Cipri C., Guastamacchia E., Triggiani V., Papini E. (2018). Shift from Levothyroxine Tablets to Liquid Formulation at Breakfast Improves Quality of Life of Hypothyroid Patients. Endocr. Metab. Immune Disord. Drug Targets.

[45] Wong C.K., Lang B.H., Lam C.L. (2016). A systematic review of quality of thyroid-specific health-related quality-of-life instruments recommends ThyPRO for patients with benign thyroid diseases. J. Clin. Epidemiol..

[46] Pollock M.A., Sturrock A., Marshall K., Davidson K.M., Kelly C.J., McMahon A.D., McLaren E.H. (2001). Thyroxine treatment in patients with symptoms of hypothyroidism but thyroid function tests within the reference range: Randomised double blind placebo controlled crossover trial. BMJ.

[47] Sawka A.M., Gerstein H.C., Marriott M.J., MacQueen G.M., Joffe R.T. (2003). Does a combination regimen of thyroxine (T4) and 3,5,3′-triiodothyronine improve depressive symptoms better than T4 alone in patients with hypothyroidism? Results of a double-blind, randomized, controlled trial. J. Clin. Endocrinol. Metab..

[48] Walsh J.P., Shiels L., Lim E.M., Bhagat C.I., Ward L.C., Stuckey B.G., Dhaliwal S.S., Chew G.T., Bhagat M.C., Cussons A.J. (2003). Combined thyroxine/liothyronine treatment does not improve well-being, quality of life, or cognitive function compared to thyroxine alone: A randomized controlled trial in patients with primary hypothyroidism. J. Clin. Endocrinol. Metab..

[49] Roos A., Linn-Rasker S.P., van Domburg R.T., Tijssen J.P., Berghout A. (2005). The starting dose of levothyroxine in primary hypothyroidism treatment: A prospective, randomized, double-blind trial. Arch. Intern. Med..

[50] Escobar-Morreale H.F., Botella-Carretero J.I., Gomez-Bueno M., Galan J.M., Barrios V., Sancho J. (2005). Thyroid hormone replacement therapy in primary hypothyroidism: A randomized trial comparing L-thyroxine plus liothyronine with L-thyroxine alone. Ann. Intern. Med..

[51] Appelhof B.C., Fliers E., Wekking E.M., Schene A.H., Huyser J., Tijssen J.G., Endert E., van Weert H.C., Wiersinga W.M. (2005). Combined therapy with levothyroxine and liothyronine in two ratios, compared with levothyroxine monotherapy in primary hypothyroidism: A double-blind, randomized, controlled clinical trial. J. Clin. Endocrinol. Metab..

[52] Walsh J.P., Ward L.C., Burke V., Bhagat C.I., Shiels L., Henley D., Gillett M.J., Gilbert R., Tanner M., Stuckey B.G. (2006). Small changes in thyroxine dosage do not produce measurable changes in hypothyroid symptoms, well-being, or quality of life: Results of a double-blind, randomized clinical trial. J. Clin. Endocrinol. Metab..

[53] Razvi S., Ingoe L., Keeka G., Oates C., McMillan C., Weaver J.U. (2007). The beneficial effect of L-thyroxine on cardiovascular risk factors, endothelial function, and quality of life in subclinical hypothyroidism: Randomized, crossover trial. J. Clin. Endocrinol. Metab..

[54] Samuels M.H., Schuff K.G., Carlson N.E., Carello P., Janowsky J.S. (2007). Health status, mood, and cognition in experimentally induced subclinical hypothyroidism. J. Clin. Endocrinol. Metab..

[55] Samuels M.H., Schuff K.G., Carlson N.E., Carello P., Janowsky J.S. (2008). Health status, mood, and cognition in experimentally induced subclinical thyrotoxicosis. J. Clin. Endocrinol. Metab..

[56] Nygaard B., Jensen E.W., Kvetny J., Jarlov A., Faber J. (2009). Effect of combination therapy with thyroxine (T4) and 3,5,3′-triiodothyronine versus T4 monotherapy in patients with hypothyroidism, a double-blind, randomised cross-over study. Eur. J. Endocrinol..

[57] Panicker V., Saravanan P., Vaidya B., Evans J., Hattersley A.T., Frayling T.M., Dayan C.M. (2009). Common variation in the DIO2 gene predicts baseline psychological well-being and response to combination thyroxine plus triiodothyronine therapy in hypothyroid patients. J. Clin. Endocrinol. Metab..

[58] Bolk N., Visser T.J., Nijman J., Jongste I.J., Tijssen J.G., Berghout A. (2010). Effects of evening vs morning levothyroxine intake: A randomized double-blind crossover trial. Arch. Intern. Med..

[59] Reuters V.S., Almeida Cde P., Teixeira Pde F., Vigario Pdos S., Ferreira M.M., Castro C.L., Brasil M.A., Costa A.J., Buescu A., Vaisman M. (2012). Effects of subclinical hypothyroidism treatment on psychiatric symptoms, muscular complaints, and quality of life. Arq. Bras. Endocrinol. Metabol..

[60] Hoang T.D., Olsen C.H., Mai V.Q., Clyde P.W., Shakir M.K. (2013). Desiccated thyroid extract compared with levothyroxine in the treatment of hypothyroidism: A randomized, double-blind, crossover study. J. Clin. Endocrinol. Metab..

[61] Stott D.J., Gussekloo J., Kearney P.M., Rodondi N., Westendorp R.G., Mooijaart S., Kean S., Quinn T.J., Sattar N., Hendry K. (2017). Study protocol; Thyroid hormone Replacement for Untreated older adults with Subclinical hypothyroidism—A randomised placebo controlled Trial (TRUST). BMC Endocr. Disord..

[62] Werneck F.Z., Coelho E.F., Almas S.P., Garcia M., Bonfante H.L.M., Lima J.R.P., Vigario P.D.S., Mainenti M.R.M., Teixeira P., Vaisman M. (2018). Exercise training improves quality of life in women with subclinical hypothyroidism: A randomized clinical trial. Arch. Endocrinol. Metab..

[63] Rezaei S., Abedi P., Maraghi E., Hamid N., Rashidi H. (2020). The effectiveness of cognitive- behavioral therapy on quality of life in women with hypothyroidism in the reproductive age: A randomized controlled trial. Thyroid Res..

[64] van der Gaag E., van der Palen J., Schaap P., van Voorthuizen M., Hummel T. (2020). A Lifestyle (Dietary) Intervention Reduces Tiredness in Children with Subclinical Hypothyroidism, a Randomized Controlled Trial. Int. J. Environ. Res. Public Health.

[65] de Montmollin M., Feller M., Beglinger S., McConnachie A., Aujesky D., Collet T.H., Ford I., Gussekloo J., Kearney P.M., McCarthy V.J.C. (2020). L-Thyroxine Therapy for Older Adults with Subclinical Hypothyroidism and Hypothyroid Symptoms: Secondary Analysis of a Randomized Trial. Ann. Intern. Med..

[66] Wekking E.M., Appelhof B.C., Fliers E., Schene A.H., Huyser J., Tijssen J.G., Wiersinga W.M. (2005). Cognitive functioning and well-being in euthyroid patients on thyroxine replacement therapy for primary hypothyroidism. Eur. J. Endocrinol..

[67] Gulseren S., Gulseren L., Hekimsoy Z., Cetinay P., Ozen C., Tokatlioglu B. (2006). Depression, anxiety, health-related quality of life, and disability in patients with overt and subclinical thyroid dysfunction. Arch. Med. Res..

[68] Samuels M.H., Schuff K.G., Carlson N.E., Carello P., Janowsky J.S. (2007). Health status, psychological symptoms, mood, and cognition in L-thyroxine-treated hypothyroid subjects. Thyroid.

[69] Quinque E.M., Villringer A., Kratzsch J., Karger S. (2013). Patient-reported outcomes in adequately treated hypothyroidism—Insights from the German versions of ThyDQoL, ThySRQ and ThyTSQ. Health Qual. Life Outcomes.

[70] Vigario Pdos S., Vaisman F., Coeli C.M., Ward L., Graf H., Carvalho G., Junior R.M., Vaisman M. (2013). Inadequate levothyroxine replacement for primary hypothyroidism is associated with poor health-related quality of life-a Brazilian multicentre study. Endocrine.

[71] Mithal A., Dharmalingam M., Tewari N. (2014). Are patients with primary hypothyroidism in India receiving appropriate thyroxine replacement? An observational study. Indian J. Endocrinol. Metab..

[72] Samuels M.H., Kolobova I., Smeraglio A., Peters D., Janowsky J.S., Schuff K.G. (2014). The effects of levothyroxine replacement or suppressive therapy on health status, mood, and cognition. J. Clin. Endocrinol. Metab..

[73] Kelderman-Bolk N., Visser T.J., Tijssen J.P., Berghout A. (2015). Quality of life in patients with primary hypothyroidism related to BMI. Eur. J. Endocrinol..

[74] Sowinski J., Sawicka-Gutaj N., Ziolkowska P., Ruchala M. (2016). Effect of free triiodothyronine concentration on the quality of life of patients treated with levothyroxine. Pol. Arch. Med. Wewn..

[75] Samuels M.H., Kolobova I., Smeraglio A., Niederhausen M., Janowsky J.S., Schuff K.G. (2016). Effect of Thyroid Function Variations Within the Laboratory Reference Range on Health Status, Mood, and Cognition in Levothyroxine-Treated Subjects. Thyroid.

[76] Jonklaas J., Burman K.D. (2016). Daily Administration of Short-Acting Liothyronine Is Associated with Significant Triiodothyronine Excursions and Fails to Alter Thyroid-Responsive Parameters. Thyroid.

[77] Rolfes L., van Hunsel F., Taxis K., van Puijenbroek E. (2016). The Impact of Experiencing Adverse Drug Reactions on the Patient’s Quality of Life: A Retrospective Cross-Sectional Study in the Netherlands. Drug Saf..

[78] Young Cho Y., Jeong Kim H., Won Jang H., Hyuk Kim T., Ki C.S., Wook Kim S., Hoon Chung J. (2017). The relationship of 19 functional polymorphisms in iodothyronine deiodinase and psychological well-being in hypothyroid patients. Endocrine.

[79] Wouters H.J., van Loon H.C., van der Klauw M.M., Elderson M.F., Slagter S.N., Kobold A.M., Kema I.P., Links T.P., van Vliet-Ostaptchouk J.V., Wolffenbuttel B.H. (2017). No Effect of the Thr92Ala Polymorphism of Deiodinase-2 on Thyroid Hormone Parameters, Health-Related Quality of Life, and Cognitive Functioning in a Large Population-Based Cohort Study. Thyroid.

[80] Lombardi C.P., Bocale R., Barini A., Barini A., D’Amore A., Boscherini M., Bellantone R. (2017). Comparative study between the effects of replacement therapy with liquid and tablet formulations of levothyroxine on mood states, self-perceived psychological well-being and thyroid hormone profile in recently thyroidectomized patients. Endocrine.

[81] Samuels M.H., Kolobova I., Niederhausen M., Janowsky J.S., Schuff K.G. (2018). Effects of Altering Levothyroxine (L-T4) Doses on Quality of Life, Mood, and Cognition in L-T4 Treated Subjects. J. Clin. Endocrinol. Metab..

[82] Michaelsson L.F., la Cour J.L., Medici B.B., Watt T., Faber J., Nygaard B. (2018). Levothyroxine/Liothyronine Combination Therapy and Quality of Life: Is It All about Weight Loss?. Eur. Thyroid J..

[83] Tariq A., Wert Y., Cheriyath P., Joshi R. (2018). Effects of Long-Term Combination LT4 and LT3 Therapy for Improving Hypothyroidism and Overall Quality of Life. South. Med. J..

[84] Sawicka-Gutaj N., Ruchala M., Feldt-Rasmussen U., Rasmussen A.K., Hegedus L., Bonnema S.J., Groenvold M., Bjorner J.B., Watt T. (2018). Patients with Benign Thyroid Diseases Experience an Impaired Sex Life. Thyroid.

[85] Akin O. (2018). Morning vs. bedtime levothyroxine administration: What is the ideal choice for children?. J. Pediatr. Endocrinol. Metab..

[86] Djurovic M., Pereira A.M., Smit J.W.A., Vasovic O., Damjanovic S., Jemuovic Z., Pavlovic D., Miljic D., Pekic S., Stojanovic M. (2018). Cognitive functioning and quality of life in patients with Hashimoto thyroiditis on long-term levothyroxine replacement. Endocrine.

[87] Karmisholt J., Andersen S. (2019). Detecting True Change in the Hospital Anxiety and Depression Scale, SF-36, and Hypothyroid Score when Monitoring Patients with Subclinical Hypothyroidism. Eur. Thyroid J..

[88] Mooijaart S.P., Du Puy R.S., Stott D.J., Kearney P.M., Rodondi N., Westendorp R.G.J., den Elzen W.P.J., Postmus I., Poortvliet R.K.E., van Heemst D. (2019). Association Between Levothyroxine Treatment and Thyroid-Related Symptoms Among Adults Aged 80 Years and Older with Subclinical Hypothyroidism. JAMA.

[89] Recker S., Voigtlander R., Viehmann A., Dunschen K., Kerp H., Frank-Raue K., Leidig-Bruckner G., Graf D., Lederbogen S., Dietrich J.W. (2019). Thyroid Related Quality of Life in Elderly with Subclinical Hypothyroidism and Improvement on Levothyroxine is Distinct from that in Young Patients (TSAGE). Horm. Metab. Res..

[90] Tan N.C., Chew R.Q., Subramanian R.C., Sankari U., Koh Y.L.E., Cho L.W. (2019). Patients on levothyroxine replacement in the community: Association between hypothyroidism symptoms, co-morbidities and their quality of life. Fam. Pract..

[91] Hirtz R., Keesen A., Holling H., Hauffa B.P., Hinney A., Grasemann C. (2020). No Effect of Thyroid Dysfunction and Autoimmunity on Health-Related Quality of Life and Mental Health in Children and Adolescents: Results from a Nationwide Cross-Sectional Study. Front. Endocrinol..

[92] Rehman M.U., Ali S.S., Khan N., Ahmad I., Ullah I. (2020). Using Thypro 39 Scale for Predicting the Quality of Life In Hypothyroid Patients At Lady Reading Hospital. JAMC.

[93] Moron-Diaz M., Saavedra P., Alberiche-Ruano M.P., Rodriguez-Perez C.A., Lopez-Plasencia Y., Marrero-Arencibia D., Gonzalez-Lleo A.M., Boronat M. (2020). Correlation between TSH levels and quality of life among subjects with well-controlled primary hypothyroidism. Endocrine.

[94] Gullo D., Latina A., Frasca F., Le Moli R., Pellegriti G., Vigneri R. (2011). Levothyroxine monotherapy cannot guarantee euthyroidism in all athyreotic patients. PLoS ONE.

[95] Peterson S.J., McAninch E.A., Bianco A.C. (2016). Is a Normal TSH Synonymous With “Euthyroidism” in Levothyroxine Monotherapy?. J. Clin. Endocrinol. Metab..

[96] Gereben B., McAninch E.A., Ribeiro M.O., Bianco A.C. (2015). Scope and limitations of iodothyronine deiodinases in hypothyroidism. Nat. Rev. Endocrinol..

[97] Ettleson M.D., Bianco A.C. (2020). Individualized Therapy for Hypothyroidism: Is T4 Enough for Everyone?. J. Clin. Endocrinol. Metab..

[98] Hennessey J.V., Espaillat R. (2018). Current evidence for the treatment of hypothyroidism with levothyroxine/levotriiodothyronine combination therapy versus levothyroxine monotherapy. Int. J. Clin. Pract..

[99] Wiersinga W.M. (2019). T4 + T3 combination therapy: Any progress?. Endocrine.

[100] Stott D.J., Rodondi N., Kearney P.M., Ford I., Westendorp R.G.J., Mooijaart S.P., Sattar N., Aubert C.E., Aujesky D., Bauer D.C. (2017). Thyroid Hormone Therapy for Older Adults with Subclinical Hypothyroidism. N. Engl. J. Med..

[101] Baldini M., Colasanti A., Orsatti A., Airaghi L., Mauri M.C., Cappellini M.D. (2009). Neuropsychological functions and metabolic aspects in subclinical hypothyroidism: The effects of L-thyroxine. Prog. Neuropsychopharmacol. Biol. Psychiatry.

[102] Watt T., Hegedus L., Bjorner J.B., Groenvold M., Bonnema S.J., Rasmussen A.K., Feldt-Rasmussen U. (2012). Is Thyroid Autoimmunity per se a Determinant of Quality of Life in Patients with Autoimmune Hypothyroidism?. Eur. Thyroid J..

[103] van der Deure W.M., Appelhof B.C., Peeters R.P., Wiersinga W.M., Wekking E.M., Huyser J., Schene A.H., Tijssen J.G., Hoogendijk W.J., Visser T.J. (2008). Polymorphisms in the brain-specific thyroid hormone transporter OATP1C1 are associated with fatigue and depression in hypothyroid patients. Clin. Endocrinol..

[104] Mitchell A.L., Hegedus L., Zarkovic M., Hickey J.L., Perros P. (2020). Patient satisfaction and quality of life in hypothyroidism: An online survey by the british thyroid foundation. Clin. Endocrinol..

[105] Watt T., Groenvold M., Rasmussen A.K., Bonnema S.J., Hegedus L., Bjorner J.B., Feldt-Rasmussen U. (2006). Quality of life in patients with benign thyroid disorders. A review. Eur. J. Endocrinol..

[106] Goichot B., Raverot V., Klein M., Vija Racaru L., Abeillon-Du Payrat J., Lairez O., Leroy R., Cailleux A., Wolff P., Groussin L. (2020). Management of thyroid dysfunctions in the elderly. French Endocrine Society consensus statement 2019. Long version. Ann. Endocrinol..

[107] Smith C.D., Grondin R., LeMaster W., Martin B., Gold B.T., Ain K.B. (2015). Reversible cognitive, motor, and driving impairments in severe hypothyroidism. Thyroid.

[108] Feller M., Snel M., Moutzouri E., Bauer D.C., de Montmollin M., Aujesky D., Ford I., Gussekloo J., Kearney P.M., Mooijaart S. (2018). Association of Thyroid Hormone Therapy With Quality of Life and Thyroid-Related Symptoms in Patients With Subclinical Hypothyroidism: A Systematic Review and Meta-analysis. JAMA.

[109] Roberts L.M., Pattison H., Roalfe A., Franklyn J., Wilson S., Hobbs F.D., Parle J.V. (2006). Is subclinical thyroid dysfunction in the elderly associated with depression or cognitive dysfunction?. Ann. Intern. Med..

[110] Pasqualetti G., Pagano G., Rengo G., Ferrara N., Monzani F. (2015). Subclinical Hypothyroidism and Cognitive Impairment: Systematic Review and Meta-Analysis. J. Clin. Endocrinol. Metab..

